# Alterations of regional homogeneity in pediatric bipolar depression: a resting-state fMRI study

**DOI:** 10.1186/s12888-014-0222-y

**Published:** 2014-08-06

**Authors:** Weijia Gao, Qing Jiao, Shaojia Lu, Yuan Zhong, Rongfeng Qi, Dali Lu, Qian Xiao, Fan Yang, Guangming Lu, Linyan Su

**Affiliations:** Mental Health Institute of The Second Xiangya Hospital, National Technology Institute of Psychiatry, Key Laboratory of Psychiatry and Mental Health of Hunan Province, Central South University, No. 139 Renmin Road, Changsha, 410011 Hunan China; Department of Child Psychology, The Children’s Hospital, Zhejiang University School of Medicine, Hangzhou, Zhejiang China; Department of Radiology, Taishan Medical University, Taian, Shandong China; Department of Mental Health, First Affiliated Hospital, Zhejiang University School of Medicine, Hangzhou, Zhejiang China; Department of Medical Imaging, Jinling Hospital, Clinical School of Medical College, Nanjing University, 305 Zhongshan East Road, Nanjing, 210002 Jiangsu China; Department of Psychology, Nanjing Normal University, Nanjing, Jiangsu China

**Keywords:** Pediatric bipolar disorder (PBD), Depression episode, Resting-fMRI, Regional homogeneity (ReHo)

## Abstract

**Background:**

Pediatric bipolar disorder (PBD) has attracted increasing attentions due to its high prevalence and great influence on social functions of children and adolescents. However, the pathophysiology underlying PBD remains unclear. In the present study, the resting-state functional magnetic resonance imaging (fMRI) was used to detect abnormalities of baseline brain functions in depressed PBD youth.

**Methods:**

Seventeen youth with PBD-depression aged 10 - 18 years old and 18 age- and sex-matched normal controls were recruited in this study. The fMRI data under resting state were obtained on a Siemens 3.0 Tesla scanner and were analyzed using the regional homogeneity (ReHo) method. Correlations between the ReHo values of each survived area and the severity of depression symptoms in patients were further analyzed.

**Results:**

As compared with the control group, PBD-depression patients showed decreased ReHo in the medial frontal gyrus, bilateral middle frontal gyrus and middle temporal gyrus, and the right putamen. Significant negative correlations of the mood and feelings questionnaire scores with mean ReHo values in the medial frontal gyrus and the right middle frontal gyrus in PBD-depression patients were observed.

**Conclusion:**

Our results suggest that extensive regions with altered baseline brain activities are existed in PBD-depression and these brain regions mainly locate in the fronto-limbic circuit and associated striatal structures. Moreover, the present findings also add to our understanding that there could be unique neuropathophysiological mechanisms underlying PBD-depression.

## Background

Bipolar disorder (BD) is one of the major psychiatric illnesses which can be appeared across all age groups including children and adults. BD which may lead to emotional and cognitive function impairments [1] is always characterized by alternating mood state between mania or hypomania, and depression. As compared with adult BD, pediatric BD (PBD) often shows more atypical symptoms and comorbidities [2]. Although mania is the hallmark characteristic of BD, much more evidence suggests depression episode make a major contribution to the disability of BD patients [3]. It has been reported that BD patients spend more time in depression [4] and depression may cause greater influence on quality of life, risk of suicide, and life-long progression [5] in BD patients. To date, plenty of studies have been directed towards etiology, neurobiology, and pathogenesis of BD, however, the mechanisms responsible underlying the development of BD remain unclear.

In the last two decades, there has been a considerable amount of fMRI studies for adult BD pointing to a relevant role of brain functional abnormalities in the pathophysiology of the disease. Most of the studies have focused on altered brain activations in the cortico-limbic pathways which are responsible for emotional regulation [6,7]. The most consistent fMRI findings in previous studies for BD are hypoactivation of the frontal lobe [8], hyperactivation of the limbic structures [9,10] and abnormal connectivity between frontal and limbic structures [11,12]. In this context, the dysregulation of mood and developments of extreme mood states in BD patients are always attributed to disruptions of prefrontal modulations of limbic structures [7].

Although many studies have focused on baseline brain functional changes in BD patients, most studies have investigated adult BD, and relatively less research on PBD has been published. To our knowledge, only four resting-state fMRI studies have focused on PBD patients. Using independent component analysis (ICA) method, Wu et al. (2013) found that PBD-mania patients revealed altered affective, executive and sensorimotor resting state networks when comparing to normal controls [13]. In two previous studies, we also found that PBD-mania patients showed abnormal resting-state neuronal activities in the basal ganglia, parietal cortex, occipital cortex [14], and the ventral-affective and dorsal-cognitive circuits [15]. Finally, in the study of Dickstein et al. (2010), significantly decreased functional connectivity in the fronto-temporal circuit was revealed in euthymic PBD patients [16]. All these studies above have provided some evidence for the resting state brain functional changes in PBD, but none of them choosing PBD-depression as subjects. This could be a huge limitation since limited studies which carefully classify the episode states of adult BD have provided evidence to prove that different mood episodes may cause different functional abnormalities of brain [17,18]. Cerullo et al. (2012) found that the right amygdala was significantly more positively correlated with the left inferior frontal gyrus during mania and with the right insula during depression in BD patients [18]. In a task-related fMRI study, Liu et al. (2012) observed that mania was associated with decreased right rostral prefrontal cortex activation to fearful and neutral faces, while depression was associated with increased left orbitofrontal cortex activation to fearful faces in BD patients [17]. All these indicate that fMRI studies in PBD-depression patients are eagerly needed.

Resting state fMRI is a fabulous way to characterize the baseline brain activities [19] without any tasks which can minimize the effect of the external stimuli [20]. Prior evidence shows that task-related changes in neural activation may just represent less than 5% of the brain’s total activity, while the majority of its resources have been used on task-independent, spontaneous neural activity, and resting state fMRI study is the best approach to evaluate it [16]. Regional homogeneity (ReHo) is a data–driven method which can be applied to process the resting state fMRI data through analyzing the temporal synchronization of the inter-regional fMRI signals of the whole brain [21]. ReHo is established on the base of that the blood oxygen level-dependent(BOLD) signal of a given voxel is temporally similar to its neighbors especially when the brain area is involved in a specific condition [22]. ReHo abnormalities (increase or decrease) may indicate the changes of temporal neural activities in the regional brain [23] and suggest an unbalanced local functionality or an uncompensatory reaction of the whole brain network [24]. This method has been widely used to elucidate the possible pathophysiological mechanisms underlying many child psychiatric disorders, such as PBD-mania [15], attention-deficit/hyperactivity disorder (ADHD) [25], and autism spectrum disorders [26].

Taken together, the aim of the present study was to explore baseline brain activity changes in PBD-depression patients by using ReHo.

## Methods

### Participants

Seventeen PBD-depression children and adolescents were recruited from the child and adolescent psychiatric clinic of the Second Xiangya Hospital of Central South University, Changsha, Hunan, P.R. China from July to December, 2012. Meanwhile, 18 age- and sex-matched healthy controls (HC) were also recruited through advertisements in public schools. The inclusion criteria for PBD-depression patients were as follows: 1) met the Diagnostic and Statistical Manual for Mental Disorders, Fourth Edition (DSM-IV) criteria for BD with current depression episode; 2) aged from 10 to 18 years old; 3) right-handedness; 4) Han nationality; 5) could follow the instructions to keep still during MRI scanning. The exclusion criteria for all participants included: 1) presence of major sensorimotor handicaps; 2) full-scale intelligence quotient (IQ) < 80; 3) contraindications to perform the MRI scan, including the presence of metallic implants, retractors or braces, and claustrophobia; 4) with other mental disorders, such as schizophrenia, anorexia or bulimia nervosa, and learning disabilities; 5) alcohol or drug dependence or abuse; 6) active medical or neurological diseases; 7) history of electroconvulsive therapy (ECT). The present study was approved by the ethic committee of the Second Xiangya Hospital of Central South University. Written informed consent forms were obtained from all the participants and their guardians.

### Procedures

#### Demographic and clinical assessments

The diagnoses were ascertained through the consensus by two board-certified child psychiatrists on the basis of clinical interviews and administrations of Schedule for Affective Disorders and Schizophrenia for School aged Children Present and Lifetime Versions (K-SADS-PL) [27]. The demographic and clinical data were collected using a self-designed questionnaire from all the participants. The Wechsler Abbreviated Scale of Intelligence (WASI) was used to assess the intellectual ability of all the patients and controls. The severity of mood symptoms was assessed using the Young Mania Rating Scale (YMRS) [28] and Mood and Feelings Questionnaire (MFQ) [29] on the day of MRI scanning. MFQ is a well- designed measure that is very widely used to assess the severity of depression symptoms in youth. It has 33 items and each item scores as 0 = never, 1 = sometimes, 2 = often. The total score, in general, is associated with severity of depressive symptoms. The Chinese version of MFQ was introduced in our study, which was translated by Cao et al. (2009) [30]. The MFQ showed good psychometric properties and good internal consistency (Cronbach’s alpha = 0.93) in a Chinese sample of 2592 middle school students [30].

#### MRI acquisition

Magnetic resonance imaging scans were performed using a Siemens 3.0-T scanner (Allegra; Siemens Medical System) at the Magnetic Resonance Center belonging to Hunan Provincial People’s Hospital. All the participants were asked to remain motionless and to keep eyes closed without thinking of anything in particular. A standard birdcage head coil was used, and the restraining foam pads were placed on two sides of the head to minimize head motion.

The regular axial three-dimensional T1-weighted images were acquired with a spoiled gradient recall sequence with the following imaging parameters: slice thickness = 1 mm, Gap = 0 mm, repetition time (TR) = 2300 ms, echo time (TE) = 2.03 ms, field of view (FOV) = 256*256 mm2, flip angle = 9°, matrix size = 256*256, Slices = 176.

An echo planar imaging sequence was used to obtain the functional images, the parameters were as follows: 30 axial slices, TR = 2000 ms, TE = 30 ms, matrix = 64 × 64, flip angle = 90°, FOV = 240*240 mm^2^, slice thickness = 4 mm, gap = 0.4 mm. A total of 250 brain volumes were collected, resulting in a total scan time of 500 s.

#### Image processing

The Statistical Parametric Mapping (SPM) 8 software package (http://www.fil.ion.ucl.ac.uk/spm) was used to preprocess the image data. The first ten images were deleted for the signal equilibration. Then the remaining images were conducted for slice acquisition correction and head motion correction. The fMRI data which had less than 1.0 mm of head motion and 1.0° of angular rotation were included. Moreover, the mean framewise displacement (FD) was computed by averaging FD_i_ from every time point for each subject [31]. There were no differences for the mean FD between groups (*t* = 0.413, *p* = 0.682) (Table 1). Then the fMRI images were normalized to the standard Montreal Neurological Institute (MNI) template provided by SPM and resample to the 3-mm isotropic voxels. A temporal filter (0.01 Hz < f < 0.08 Hz) were used to reduce the low-frequency drift and physiological high frequency respiratory and cardiac noise [21,32].

**Table 1:** **Demographic and clinical characteristics of all subjects (n = 35)**

	**PBD-depression n = 17 means(SD)**	**HC n = 18 means (SD)**	**t/** ***χ*** ^***2***^	***P*** **-values**
Age	14.4(1.77)	14.1(1.61)	0.527	0.602
Gender (Male/Female)	7/10	6/12	0.230	0.631
IQ	102(14.6)	105(7.72)	-0.808	0.425
YMRS score	4.18(1.47)	3.67(2.11)	0.824	0.416
MFQ score	30.3(9.67)	6.39(3.36)	9.878	0.000
Mean FD	0.128(0.03)	0.133(0.05)	0.413	0.682
Illness duration (months)	10.5(6.55)			
Onset age (years)	13.5(1.81)			
Episode times	3.88(2.53)			
Medication, n (%)				
None	9(52.9)			
Lithium	4(23.5)			
Valproate	4(23.5)			
Antipsychotic drugs	8(47.1)			
Antidepressant	2(11.8)			
Comorbidity, n (%)				
Anxiety	1(5.88)			
ADHD	2(11.8)			
OCD	1 (5.88)			

ADHD, attention deficit hyperactivity disorder; FD, framewise displacement; IQ, intelligence quotient; MFQ, mood and feelings questionnaire; HC, healthy controls; OCD, obsessive compulsive disorder; PBD, pediatric bipolar disorder; YMRS, Young Mania Rating Scale.

The acquisition of individual ReHo map was performed with REST software (http://www.resting-fmri.sourceforge.net). The Kendall’s coefficient of concordance (KCC) was calculated to measure regional homogeneity of the time series of a given voxel with its nearest neighbor (26 voxels) in a voxel-wise way that had described by Zang et al. (2004) [22]. Then a mask (made from the MNI template to assure matching with the normalization step), in the REST software, was used to remove non-brain tissue and for standardization purposes. Each individual ReHo map was divided by its own global mean KCC value within the mask [33]. Finally, the data was smoothed with a Gaussian kernel of 4 mm full-width at half-maximum.

### Statistical analysis

Between group differences in demographic variables were examined using independent two-sample *t* tests for continuous variables and chi-square tests for categorical variables in Statistical Package for the Social Sciences (SPSS) version 16.0 (SPSS Inc., Chicago, IL, USA). The level of two-tailed statistical significance was set at *p* < 0.05 for all tests.

To explore ReHo differences between PBD-depression patients and normal controls, a second-level random-effect two-sample *t* test was performed in a voxel-by-voxel manner. Significant differences were set at a corrected significance level of *p* < 0.05 [combined height threshold *p* < 0.01 (T > 2.46) and a minimum cluster size of 18 voxels]. Threshold correction was performed by using AlphaSim program (parameters were as follows: individual voxel *p* = 0.01, 1000 simulations, FWHM = 4 mm, with mask) in the REST software, which applied Monte Carlo simulation to calculate the probability of false-positive detection by taking into consideration both the individual voxel probability thresholding and cluster size [34].

Furthermore, Pearson’s correlation analyses were performed to explore the relationships between MFQ scores and mean ReHo values within regions displaying significant between-group differences in PBD-depression.

## Results

### Demographic and clinical characteristics

Demographic and clinical features of patients and healthy controls in the sample are presented in Table 1. There was no significant difference between two groups for age, gender, educational level, IQ and YMRS score. As expected, the mean MFQ score was significant higher in PBD-depression patients than controls. Nine(52.9%) patients were free of medication at the time of MRI scanning; four(23.5%) patients had comorbid disorder, particularly ADHD.

### Between-group ReHo differences

**Figure 1:**
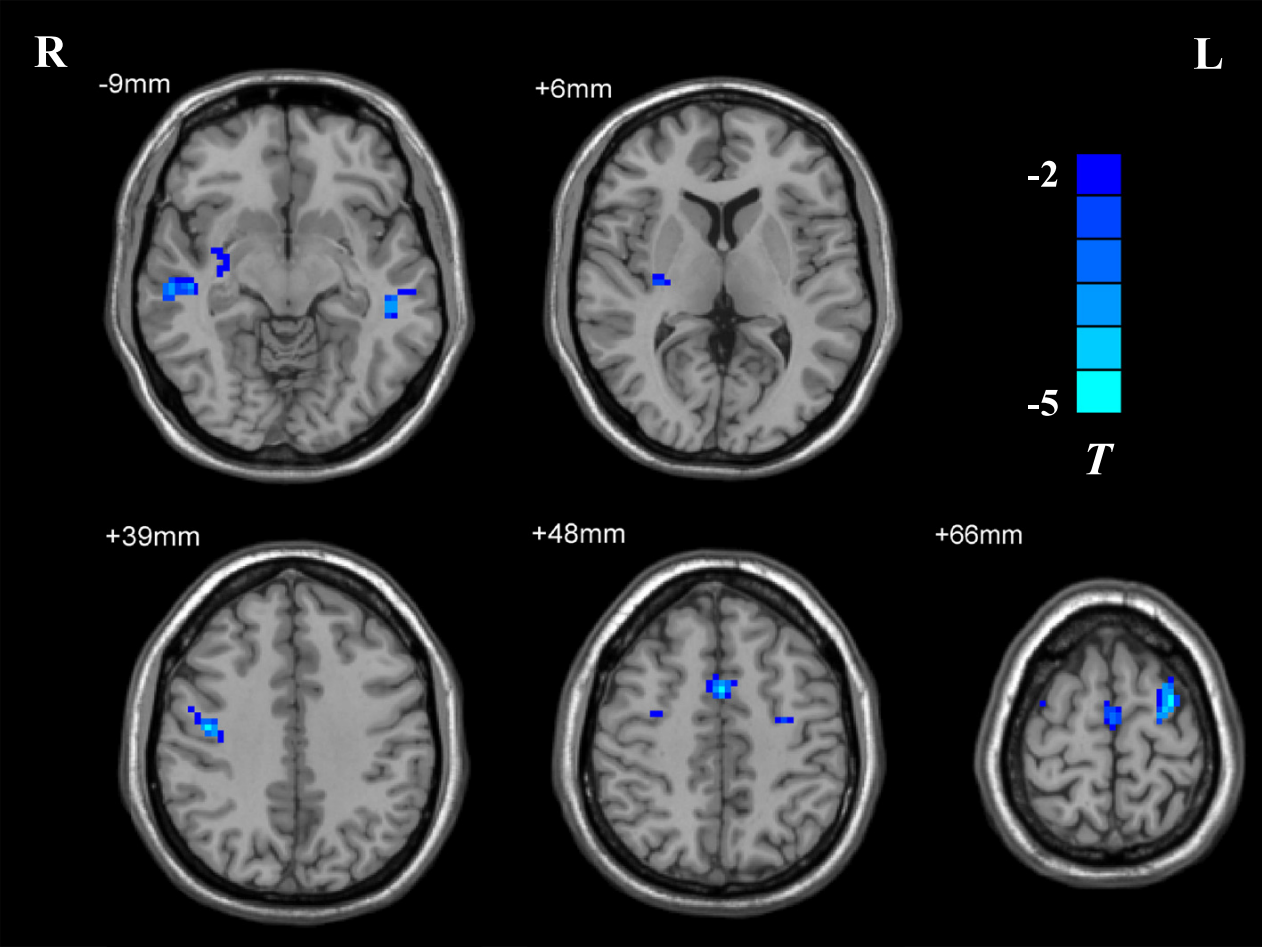
**T-statistical different maps between PBD-depression patients and healthy controls (two-sample** ***t*** **test;** ***p*** **< 0.05, AlphaSim corrected).** Regions with decreased ReHo values are shown in blue.

As compared with the control group, PBD-depression patients showed significant decreased ReHo values in the medial frontal gyrus, bilateral middle frontal gyrus and middle temporal gyrus, and the right putamen (see Table 2 and Figure 1). However, there were no brain regions revealing increased ReHo values in patients.

**Table 2:** **Brain regions showing decreased ReHo between PBD-depression patients and normal controls**

**Brain regions**	**Hemisphere**	**Cluster size**	**MNI coordinates**			***T*** **value**
			***x***	***y***	***z***	
Medial frontal gyrus		74	0	6	48	-4.98
Middle frontal gyrus	Left	95	-30	-3	66	-5.43
	Right	70	42	-12	39	-5.30
Middle temporal gyrus	Left	41	-48	-30	-9	-4.07
	Right	34	57	-21	-9	-3.90
Putamen	Right	30	30	-18	6	-3.63

MNI, Montreal Neurological Institute.cp.

### Correlations

**Figure 2:**
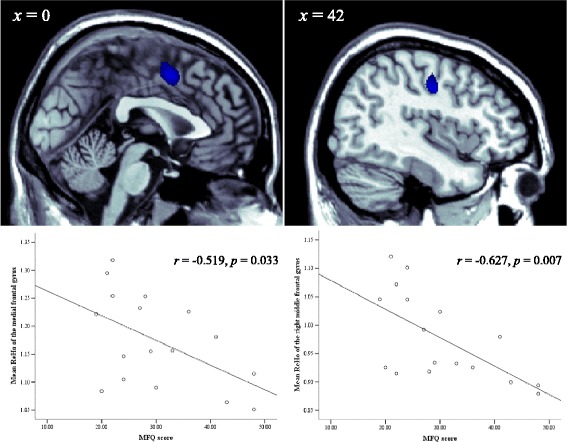
**Correlation analysis between MFQ Scores and ReHo values in the PBD-depression patients (** ***p*** **< 0.05, corrected).**

Within the PBD-depression patients, correlations of ReHo values in brain regions with significant between-group differences and MFQ scores were evaluated. Significant negative correlations were observed in the medial frontal gyrus (*r* = -0.519, *p* = 0.033) and the right middle frontal gyrus (*r* = -0.627, *p* = 0.007) (see Figure 2).

## Discussion

In the present study, ReHo was used to show neural synchronization of local brain areas in PBD-depression patients during resting state. The current results suggest that diffuse brain regions with decreased ReHo values occur in PBD-depression. These regions are mainly distributed over the medial frontal gyrus, bilateral middle frontal gyrus and middle temporal gyrus, and the right putamen. Furthermore, in PBD-depression patients, there were significant negative correlations between MFQ scores and mean ReHo values in medial frontal gyrus and right middle frontal gyrus. Our findings showed that the abnormalities of baseline brain activities in PBD-depression are much more robust than those reported in adult BD-depression [35], which may indicate that BD with early-onset could be a more severe clinic form. Consistent with our assumptions, the present findings are partly different from what we have found in our prior study for PBD-mania [15], suggesting that there could be different brain functional alterations involving in different mood episodes.

In the present study, PBD-depression patients showed decreased ReHo values in frontal regions, including the medial frontal gyrus and the bilateral middle frontal gyrus. It has been reported that the medial prefrontal cortex (mPFC) plays an important role in the regulation and generation of emotion [36], which is based on the dense and reciprocal connectivity with subcortical regions, such as amygdala [37]. Previous studies have suggested the involvement of the medial frontal gyrus in the pathophysiology of BD. Our results are consistent with previous findings showing significantly decreased absolute regional metabolism in the medial frontal gyrus [38] and increased ReHo values in the left media frontal gyrus [35] in adult BD-depression patients. In addition, the present findings are also in agreement with a study that reported decreased connectivity in the mPFC in adult BD patients (13 with mania episode and 4 with mixed episode) [39].

A second noteworthy finding was the decreased ReHo values in bilateral middle frontal gyrus. The middle frontal gyrus has been reported to be involved in decision making, affective modulation, and conflict resolution [40,41]. In a prior study for patients with first-episode BD-mania, the middle frontal gyrus was abnormally activated following a response inhibition task, which suggested that the recruitment of the middle frontal gyrus might be considered as a compensatory neural strategy to manage the demands of this task [42]. In another positron emission tomography (PET) study for medication-free outpatients with BD-depression, decreased metabolism in bilateral middle frontal gyrus was negatively correlated with the severity of depression symptoms [38]. Moreover, structural MRI studies also demonstrated that gray matter volume was reduced in the right middle frontal gyrus in a group of patients with BD II [43]. Together with our results, these findings indicate that functional abnormality in the frontal gyrus is existed not only in adult BD but also in PBD. We propose that brain functional impairments of the frontal gyrus may exist in the early stage of BD and persist into adulthood.

Decreased ReHo was also found in bilateral middle temporal gyrus in patients with PBD-depression. The temporal lobe is a visual and auditory-related brain region that plays a vital role in working memory processing and facial emotion processing [44,45]. Structural and functional MRI studies have accumulated the evidence that BD is associated with the alterations in temporal lobe. Chen et al. (2007) [46] observed decreased gray matter volume in the left middle superior temporal gyrus in adult BD patients. In addition, volume reductions in the left superior and middle temporal gyrus in adult patients with BD II were reported by another study [47]. For functional studies, adult BD-depression showed significantly lowered regional cerebral blood flow in the anterior temporal regions bilaterally [48]. Decreased metabolism in the middle temporal gyrus was significantly associated with depression symptoms in a sample of medication-free patients with adult BD-depression [38]. Another resting-state study for adult BD-depression patients noted that the amplitude of low-frequency fluctuation (ALFF) was increased in bilateral temporal gyrus in the patient group [5]. Our result, in combination with these prior findings, suggests that the middle temporal gyrus is presumably part of a relevant functional network associated with BD, especially during a depressive episode.

The final region we found to show decreased ReHo was the right putamen. Putamen belongs to the lateral paralimbic system which is involved in the motivational processes and has connectivity to the mPFC [36,49]. Abnormal brain activity in the putamen has been reported in the task-related MRI studies in BD. Blumberg et al. (2003) [50] observed increased activation of the left putamen in ten adolescents with BD while they were performing an event-related fMRI color naming Stroop task. Consistent with this finding, Surguladze et al. (2010) [51] also found increased left putamen activity in response to moderate fear and high intensity happy faces in adult BD patients as compared with normal controls. Interestingly, an adult study including medication-free BD patients (stratify groups by depression, euthymia, and mania episode) performing a negative facial emotion matching task showed greater activations in the putamen in all three BD groups when comparing to controls [52]. Of note, even in symptom-free youths at a familial risk for BD, this difference mentioned above has already occurred, which may indicate that brain functional changes in the putamen is a notably sensitive marker for early stage of BD [53]. Also functional abnormalities in the putamen may be considered as a clinic marker for early identification of PBD.

## Limitations

Several limitations should be considered when interpreting the present results. First of all, the sample size in our study is relatively small, which may give rise to the problem that some subtle changes in the brain cannot be observed. Secondly, nearly half of the patients are taking medication when scanning, thus, medication exposure becomes a potential confound in this study. Thirdly, these results should be tested in a study which compares brain functional abnormalities of a same person with different mood states.

## Conclusions

In conclusion, our results suggest that extensive regions with altered baseline brain activities are existed in PBD-depression and these brain regions mainly locate in the fronto-limbic circuit and associated striatal structures. Moreover, the present findings also add to our understanding that there could be unique neuropathophysiological mechanisms underlying PBD-depression.
